# Descriptive Study of Patients With Upper Limb Amputation As Possible Candidates for a Hand Transplant in Medellín, Colombia

**DOI:** 10.7759/cureus.22527

**Published:** 2022-02-23

**Authors:** Evert Armando Jiménez Cotes, Adolfo Alejandro López Rios, Vanesa Vásquez Sañudo, Esteban Cardona González

**Affiliations:** 1 Plastic and Reconstructive Surgery, Universidad de Antioquia, Medellín, COL; 2 Plastic and Reconstructive Surgery, Hospital Universitario San Vicente Fundación, Medellín, COL; 3 General Medicine, Universidad de Antioquia, Medellín, COL; 4 Plastic and Reconstructive Surgery, Medical Tower El Tesoro Mall, Medellín, COL

**Keywords:** upper extremity, amputation, hand injuries, hand transplant, hand

## Abstract

Introduction

The amputation of the upper limb, especially of the hand, is a serious trauma with significant functional and psychological impact. Treatment options include adaptation to the stump, use of prostheses, and composite tissue transplantation. Unfortunately, in Colombia, to date, there are no epidemiological data that characterize the sociodemographic and clinical variables of patients with upper limb amputation.

Objective

To describe the clinical and epidemiological characteristics of patients with upper limb amputation in two hospital institutions in the city of Medellín during the period 2018-2019 as a basis for developing a hand transplant program in Colombia.

Methods

A descriptive cross-sectional study was carried out where a total of 443 medical records were reviewed, of which eight records that met the inclusion criteria were identified. Variables such as age, etiology of amputation, level of amputation, comorbidities, complications, and rehabilitation process were analyzed. The information was collected in the REDCap program, and a descriptive analysis was carried out with the data obtained.

Results

From a sample of 443 amputee patients, eight were selected that met the inclusion criteria. There were 7/8 men (87%) aged 43 years. A total of 75% came from rural areas. In 3/8 patients, there was amputation of the dominant limb. The most common mechanisms were shear trauma and crushing in the context of an occupational accident. A total of 50% had a complete extra-hospital amputation, and reimplantation was not attempted in any of the cases. The most frequent levels of amputation were the proximal and distal third of the forearm. The longest follow-up time was 12 months. Only two patients mentioned the possibility of using a prosthesis during rehabilitation.

Conclusions

Three young patients were identified, without comorbidities, with amputation of the dominant limb in the context of an occupational accident and without the possibility of rehabilitation with prostheses who benefit from a possible future hand transplant. However, it is necessary to implement a composite tissue transplantation program and public health policies that allow this procedure to be performed in Colombia.

## Introduction

The amputation of the upper limb is a serious trauma with significant functional and psychological impact, which generates serious sequelae, loss of working life, high costs to the health system, and complex rehabilitation processes. In the United States, the prevalence of amputations was 1.6 million in 2005, with projections that it could double by 2050 [1]. The main cause of acquired amputations is trauma, which accounts for 80% of amputations that occur in men between the ages of 15 and 45 [2]. In Colombia, armed conflict, violence, and accidents contribute to a considerable number of patients with musculoskeletal trauma. According to the observatory for victims of the armed conflict and demining in Colombia, up to April 2021, there have been 12,053 victims of antipersonnel mines and unexploded ammunition. Out of these, 20% have died due to the accident, that is, 1 in 5 patients [3]. A total of 97% of these events occur in rural areas where people depend on manual skills to survive. Antioquia is the department with the highest number of civilian victims [3].

In Colombia, there are no epidemiological data related to hand amputation. However, it is presumed that violence, gunpowder explosions, electrical burns, and workplace accidents account for most cases.

After the amputation of the upper limb, a complex process of rehabilitation follows that requires a multidisciplinary approach to ensure reintegration to social life, adaptation to trauma, and sometimes the return to work of patients. Among the therapeutic options for the rehabilitation and reconstruction of the lost limb is the use of prostheses, which have been considered the standard of management of amputee patients. However, they have the limitation of their high cost, problems with the hygiene of the limb, the need for maintenance and frequent change of the device, and the poor aesthetic acceptance by patients, with abandonment rates of 20% [4]. In search of more acceptable therapeutic alternatives for patients with severe upper limb trauma, cadaveric donor hand transplantation has been performed since 1999, becoming a therapeutic reality for patients, largely restoring functionality and improving patients' quality of life. The first successful case was performed in the United States in Louisville, Kentucky, and is currently the hand transplant with the longest long-term follow-up with good functionality and few episodes of acute rejection [5]. After an upper limb transplant, 75% of patients recover their working life and become independent, improving their quality of life.

The indications for hand transplantation remain a matter of debate because it is a procedure that is not considered vital and carries the risks of lifelong immunosuppressive therapy. In cases of bilateral hand amputation, need for simultaneous transplantation, or unilateral amputation of the dominant limb, long-term immunosuppression is justified, with superior results compared to the use of prostheses, especially in amputations below the elbow [6]. Most amputees are not candidates for a hand transplant due to limitations in immunosuppressive therapy, clinical and surgical characteristics of the amputee segment, psychological conditions of the patient, and the need for great financial support.

In Colombia and South America, the first-hand transplant has not been performed for different reasons, including the absence of epidemiological data on the sequelae of upper limb amputation, the lack of standardized management programs and protocols, and high costs in a system of health with economic problems. However, being able to characterize the population with an upper limb amputation is the first step in developing care programs for amputee patients. These policies promote the prevention of this sequela and, in the future, will be able to offer management alternatives such as transplantation of composite fabrics.

The objective of this study is to have information on the population of patients in Medellín with upper limb amputation as an initial step to realize if it is pertinent to create an upper limb transplant program.

## Materials and methods

Type of study

A descriptive, cross-sectional study of patients with upper limb amputation who consulted between January 1, 2018, and December 31, 2019, was carried out at two institutions in the city of Medellín; the Hospital San Vicente Fundación (HSVF) and the Clínica IPS Universitaria León XIII.

Inclusion criteria

Patients between 18 and 60 years of age with a diagnosis according to the International Classification of Diseases (ICD-10) code of acute amputation from the supracondylar region of the humerus to the carpus, registered in the outpatient and hospital clinic at two hospitals in the city of Medellín.

Exclusion criteria

Patients with incomplete clinical information, amputations at the metacarpophalangeal level, and patients with old amputations were excluded.

Sample size

No sample size calculation was performed since all the stories that met the inclusion criteria were included.

Variables

A description was made of the sociodemographic characteristics, comorbidities, etiology, level of amputation, initial management, complications, rehabilitation, and postoperative follow-up of the patients who required upper limb amputation in the study period.

Collection, processing, and analysis techniques

With prior approval of the research and bioethics committee of the participating institutions, a review of the ICD-10 codes related to upper limb amputation was carried out. The medical records were analyzed by two investigators individually, who selected the medical records that met the inclusion criteria. The data of interest were registered in the REDcap® database, in which the information was collected and subsequently analyzed by the four researchers. In addition, a descriptive analysis was carried out with the Epidat version 4.2 epidemiological data analysis program.

Ethical considerations

According to Resolution 8430 of 1993, issued by the Ministry of Health of the Republic of Colombia, it is classified as a risk-free study.

## Results

We retrospectively identified 443 patients with a diagnosis of complex trauma and upper limb amputation according to ICD-10 codes admitted between January 1, 2018 and December 31, 2019, in both institutions, 346 in the HSVF and 97 in the IPS León XIII. From the designated codes, diagnoses were ruled out, excluding 435 records, and a sample of 8 medical records that met the inclusion criteria was selected.

The characteristics of the eight patients included in the analysis are shown in Table 1. It was found that 7/8 were men (87%), with a mean age of 43 years. Regarding origin, 6/8 (75%) were from rural areas. The prevailing education level was basic secondary in 5/8 (62%). Regarding profession, 2/8 were unemployed patients, 2/8 were farmers, and each of the remaining was dedicated to jobs such as mining, welder, electrician, and employee in a food company.

**Table 1:** Features of the patients. RQ: Research question; PSA: Prostate-specific antigen.

Features	Patients (N = 8)
Demographics	
Age in years (mean and RQ)	43 (27)
Men: No. (%)	7 (87%)
Rural origin: No. (%)	6 (75%)
Cigarette smoking or PSA: No. (%)	3 (37%)
Diabetes/Coronary heart disease: No. (%)	1 (12%)
Work accident: No. (%)	5 (62%)
Car accident: No. (%)	1 (12%)
Violence/Agression: No. (%)	1 (12%)
Another accident: No. (%)	1 (12%)
Clinical features	
Laterality of amputation	
Right upper limb: No. (%)	4 (50%)
Left upper limb: No. (%)	4 (50%)
Amputation of dominant limb: No. (%)	3 (37%)
ASA classification	
ASA I: No. (%)	3 ( 37%)
ASA II: No. (%)	4 (50% )
ASA III - no. (%)	1 (12%)
Cutting trauma: No. (%)	3 (37%)
Gunpowder trauma: No. (%)	1 (12%)
Crush trauma: No. (%)	3 (37%)
Electric burn: No. (%)	1 (12%)
Level of amputation	
Carpus/Wrist: No. (%)	1 (12%)
Distal third of forearm- no. (%)	3 (37%)
Proximal third or forearm: No. (%)	3 (37%)
Elbow/Supracondylar humerus: No. (%)	1 (12%)
Complications	
Infection/Osteomyelitis	2 (25%)
Rehabilitation	
Evaluation by physiatry	3 (37%)
Prosthesis prescription	2 (25%)
* American Society of Anesthesiologists (ASA)	

Regarding the clinical characteristics, 50% of the patients presented amputation of the right limb, and of all amputees, 37% presented amputation of the dominant limb. The most prevalent comorbidity was cigarette smoking in 3/8 patients, and only one of these three smoking patients also had coronary heart disease and diabetes; the rest of the patients did not present any 5/8 comorbidity (62%). A total of 50% of the patients were classified as American Society Classification (ASA) II, 37% ASA I, and only one patient was ASA III.

The most common mechanisms were cutting trauma and crushing. Both were present in 3/8 patients. The remaining patients suffered an electrical burn and gunpowder/explosive trauma. The most frequent scenario where the trauma occurred was an occupational accident with 5/8 (62%) of the patients; only one was due to aggression or violence. Half of the 4/8 patients (50%) had a complete extra-hospital amputation, and reimplantation was not attempted in any of the cases.

The most frequent levels of amputation were the proximal third and the distal third of the forearm, both with 3/8 patients; the other levels of amputation were at the carpal/wrist level and the elbow or supracondylar humerus. Orthopedics performed the amputation in 6/8 patients. In the other two, it was performed by plastic surgery at the level of the carpus/wrist and the distal third of the forearm. During hospitalization, the patient who required the most surgeries underwent three surgical procedures (two surgical scrubs and one residual limb remodeling), three patients required two interventions, and four patients required a single intervention. In addition, three patients presented with local complications, 2/8 (25%) from infection, and one with stump osteomyelitis.

Regarding hospital care, the longest hospital stay was 40 days, and the shortest stay was two days. All types of discharge were due to medical discharge. The longest follow-up time was 12 months and the shortest was four days. Three patients had an in-hospital or outpatient evaluation for physiatry, two were not evaluated, and in the remaining three, no information was found; the maximum follow-up time of 15 months. In two patients, the possibility of rehabilitation with a prosthesis was mentioned; however, there are no data in the medical history to verify the use and adherence to this device.

## Discussion

This study was allowed for the first time in Colombia to characterize patients with upper limb amputation during the 2018-2019 period in two hospital institutions in the city of Medellín. Of the 443 medical records analyzed, eight patients with acute traumatic amputation of the upper limb were identified, corresponding to 1.8% of the ICD-10 codes related to amputation. Our findings are similar to those reported in the local literature, where less than 2% of patients with upper extremity trauma present amputation [7]. The vast majority were men with a mean age of 42 years, which is consistent with reported epidemiological data showing that most cases occur at active working ages [8]. The most frequent etiology was traumatic in the context of an occupational accident and from rural areas where people depend on manual skills to survive. The other mechanisms found were electrical burn and gunpowder/explosives trauma. Although in Colombia, it is presumed that armed conflict, violence, and accidents contribute to a considerable number of cases, this was not evidenced in our analysis. It was most likely related to the study period and the decrease in the number of wounded in the context of armed conflicts in Colombia due to the peace process with illegal armed groups [9].

The morbidity associated with the upper limb amputation is greater than that reported in the lower limb. This is explained by the drastic change in daily life activities without the use of the hand, as well as the difficulty reported with the use of prosthesis of the upper limb [10]. In our study, 37% of the patients had amputation of the dominant limb, which can significantly impact their quality of life. In this study, no laterality of the amputated limb predominated, which differs from other studies where amputation of the left upper limb predominates [8-11]. The most common anatomical level of amputation was the proximal third and distal third of the forearm.

In the study by Benitez MB and Posada SG, 415 amputations were found at any level of the upper limb. The most common level of amputation was the hand, followed by the forearm, and lastly, the arm without specifying the level [7].

Remarkably, none of the patients in our study attempted reimplantation of the amputated segment. Despite being Medellín the place where hand reimplantation was performed for the first time in Latin America and having long experience at the local level in this procedure, the reimplantation attempt was not documented, most likely due to limitations in transport and preservation of the segment amputee, the absence of an upper limb reimplantation network, and the availability of surgeons trained in this procedure.

Regarding the rehabilitation process, the possibility of rehabilitation with a prosthesis was mentioned in two patients; however, there are no data in the clinical history to verify the use and adherence to this device. Therefore, the patients identified in this study meet the favorable characteristics for a possible future hand transplant: the loss of the dominant limb in a young population, without systemic or mental illnesses, and without the possibility of rehabilitation with prostheses. Furthermore, this procedure has presented significant advances in microsurgical techniques and immunosuppressive treatment during the last 20 years, which has allowed obtaining transplant survival rates of up to 98% in the cases of isolated unilateral or bilateral hand transplants [12]. Currently, more than 107 transplants have been performed worldwide, with good aesthetic and functional results due to their similar appearance, weight, texture, and intuitive control [13]. Unlike solid organ transplants, hand transplantation is not a vital surgery, but it does manage to regain functionality by improving the quality of life of patients [14,15].

The limitations of this article are the size of the sample, the heterogeneity of the data in terms of demographic characteristics, etiology of the trauma, level of amputation, dominance, among others. The lack of local epidemiological data allows us to contrast our results with what has been published in the literature.

## Conclusions

The amputation of the upper limb is a catastrophic event that fortunately occurs in a low percentage in our city, although with clinical characteristics very similar to those reported worldwide. In our study, the vast majority of amputees were young, in the context of an occupational accident and with amputation of the dominant limb in 37% of cases. These patients would be possible candidates for a future hand transplant; however, it is necessary to implement care and rehabilitation programs for the amputee patient to ensure optimal postoperative results and return to work life.
